# Obituary: Professor Keith Rayner (1943–2015)

**DOI:** 10.3389/fpsyg.2015.00393

**Published:** 2015-04-09

**Authors:** Victor S. Ferreira

**Affiliations:** Department of Psychology, University of CaliforniaSan Diego, La Jolla, CA, USA

**Keywords:** obituary, visual attention, eye-tracking, biography

Distinguished Professor Keith Rayner, Atkinson Family Chair of Psychology at UC San Diego, passed away on January 21, 2015. He was 71 years old.

Keith was born June 20th, 1943, in Dover, England. His family immigrated to the United States in 1949, and settled in Salt Lake City, Utah. He received Bachelor's and Master's degrees from the University of Utah before earning a Ph.D. from Cornell University in 1974. Keith began his career at the University of Rochester in 1973. He moved to the University of Massachusetts in 1978, where he became Distinguished University Professor in 1991. In 2008, he was recruited to UC San Diego as the Atkinson Family Chair in Psychology.

Keith received many awards for his distinguished career. He delivered the Bartlett Lecture in recognition of a Lifetime Achievement Award from the Experimental Psychology Society. He also received the William James Award for Lifetime Achievement from the Association for Psychological Science, the Division 3 Lifetime Achievement Award from the American Psychological Association, the Lifetime Achievement Award from the American Literacy Association, a research award from the Federation of Associations in Behavioral and Brain Sciences, and an Outstanding Mentor Award from Women in Cognitive Science. He was a Chinese Academy of Sciences Distinguished Visiting Professor, and the Carnegie Centennial Professor for Scotland.

Keith was the world's leading expert in the study of the cognitive mechanisms underlying the ability to read in children and adults. In the 1970s, he pioneered the use of eye-tracking methodology in psychological research, which enabled the precise measurement and manipulation of read text with millisecond control. Through Keith's work, researchers can accurately predict how long an adult will look at each word on a page as they engage in the skilled reading of natural text, as accounted for by the E–Z Reader Model, developed with his colleagues and students. In addition to illuminating our understanding of the operation of a critical human skill, this research has significant promise for realistically improving adult reading performance and for the teaching of skilled reading to children.

Keith published over 400 peer-reviewed research articles across his career, including over 130 since his arrival at UC San Diego in 2008. He co-wrote *The Psychology of Reading* in 1989, and edited or co-edited 10 volumes of research on reading and eye movements. His research was continuously funded across his career by the National Institutes of Health, and he received funding from the National Science Foundation, Microsoft Research, and the Office of Naval Research.

One of Keith's proudest achievements was his record of training and placement of graduate students and post-doctoral researchers he mentored. He has placed 19 graduate students and 25 post-doctoral researchers in faculty positions around the world, as well as four graduate students and four post-doctoral researchers in professional positions in industry and government.

Keith left a deep mark on his colleagues in his discipline and at the University of Massachusetts and at UC San Diego, as well as the dozens of students and scholars he has mentored throughout the world across his 40-year career. He was an avid sports fan, especially of the Boston Red Sox, the New England Patriots, and the Boston Celtics. He was an active member of the LDS church and served in various capacities throughout his life. He is survived by his wife Susan, his children, Ashley (Jason) and Jonathan (Becky), his two granddaughters, Isabel and Samantha, his mother Olive, siblings Pete (Kathy), Sue (Brent), and Julie.

A ceremony in honor of Keith's academic accomplishments will be held in the coming months and will be announced separately. Colleagues and friends can express memories of Keith at http://www.forevermissed.com/keith-rayner/. In lieu of flowers, contributions can be made to the Keith Rayner Memorial Award, to which contributions can be made at https://giveto.ucsd.edu/make-a-gift?id=1b28a2d1-db81-43c9-bc84-37a99b44fff6.

## Conflict of interest statement

The author declares that the research was conducted in the absence of any commercial or financial relationships that could be construed as a potential conflict of interest.

